# Molecular identification and epidemiological comparison of *Cryptosporidium* spp. among different pig breeds in Tibet and Henan, China

**DOI:** 10.1186/s12917-019-1847-3

**Published:** 2019-03-28

**Authors:** Shuangjian Zheng, Dongfang Li, Chunxiang Zhou, Sumei Zhang, Yayun Wu, Yankai Chang, Yuancai Chen, Jianying Huang, Changshen Ning, Gaiping Zhang, Longxian Zhang

**Affiliations:** 1 College of Animal Science and Veterinary Medicine, Henan Agricultural University, Zhengzhou, 450002 People’s Republic of China; 2 College of Veterinary Medicine, Yangzhou University, Yangzhou, 225009 China

**Keywords:** China, *Cryptosporidium*, Pig, Zoonotic

## Abstract

**Background:**

*Cryptosporidium* spp. are important zoonotic pathogens infecting a wide range of vertebrate hosts, and causing moderate to severe diarrhea in humans. *Cryptosporidium* infections are frequently reported in humans and animals worldwide, but little research has been done on local pig breeds such as Tibetan pigs and Yunan Black pigs and imported pig breeds such as Landrace pigs in China. Therefore, a total of 1089 pig fecal samples from four intensive farms in four areas of China, including Tibetan pigs from Gongbujiangda County (*n* = 180) and Mainling County (*n* = 434), Tibet, Yunan Black pigs from Sanmenxia, Henan Province (*n* = 246), and Landrace pigs from Kaifeng, Henan Province (*n* = 229), and were screened for the presence of *Cryptosporidium* with microscopy and nested PCR amplification of the small subunit rRNA gene.

**Results:**

The total infection rate of *Cryptosporidium* in 1089 fecal samples of three different pig breeds was 2.11% (23/1089), and the infection rates of Tibetan pigs, Yunan Black pigs, and Landrace pigs were 0.49% (3/614), 0.41% (1/246), and 8.30% (19/229), respectively. The prevalence of *Cryptosporidium* infection was significantly higher in weaned piglets (1–2 months) (4.36%, 21/482) than in younger and older age groups (*p* < 0.01). Sequence analysis of positive samples revealed that there was no mixed infection in our study population, which included 12 cases of *C. suis* mono-infections (52.17%, 12/23) and 11 cases of *C. scrofarum* mono-infections (47.83%, 11/23). *C. suis* was identified in one pre-weaned piglet (< 1 month) and 11 weaned piglets (1–2 months), while *C. scrofarum* was only detected in 10 weaned piglets (1–2 months) and one finished pig (> 2 months).

**Conclusions:**

This is the first report on the identification of *Cryptosporidium* spp. in Tibetan pigs, and our findings also elucidate the occurrence and distribution of *Cryptosporidium* in three different pig breeds in Tibet and Henan, China. More molecular epidemiological studies are required to better clarify the prevalence and public health significance of *Cryptosporidium* in different pigs.

## Background

*Cryptosporidium* is an important parasitic pathogen, generally causing self-limiting diarrhea in livestock, wild animals, and humans. However, it may cause severe debilitating disease in immunocompromised patients, especially those with acquired immune deficiency syndrome. The pathogen is transmitted via the fecal-oral route in both humans and animals, usually through the ingestion of contaminated water or food.

There is an extensive genetic variation within the genus *Cryptosporidium*, with 37 valid *Cryptosporidium* species recognized to date [1–4]. Of these, *C. suis*, *C. scrofarum* (formerly *Cryptosporidium* pig genotype II), *C. parvum*, *C. muris*, *C. tyzzeri* (formerly *Cryptosporidium* mouse genotype I), and *C. andersoni* have been identified from pigs [5, 6]. There are also reports that both *C. hominis* and *C. meleagridis* can infect pigs [7–9]. Interestingly, cases of *C. suis* and *C. scrofarum* infection have been reported in humans in recent years, suggesting that these two pig-adapted *Cryptosporidium* species are potentially zoonotic [10–12]. *C. suis* was first identified in a 24-year old HIV patient in Peru in 2002 [13], and was then identified in HIV patients in Peru and China in 2007 and 2013, respectively [14, 15]. *C. suis* has also been found in patients with digestive system ailments in the United Kingdom and Madagascar [12, 16]. At present, there has only been one reported case of *C. scrofarum* infection in humans, which occurred in the Czech Republic in 2009 [17].

Landrace pigs are very famous in the world, mainly because of its good reproductive performance and fast growth, but have poor stress resistance [18]. Some indigenous Chinese pig breeds have lower growth rates and lean meat content, compared with conventional western pig breeds, but they are better able to survive in harsh environments. Yunan Black pigs, whose mother is one of some excellent local pig breeds in Henan, were bred by introducing Duroc pigs lineages in a large proportion (62.5%). Its main characteristics are slow growth but good meat quality. Tibetan pigs, also known as ginseng pigs, are mainly found in the eastern region of the Qinghai-Tibet Plateau, the northwest of Yunnan Province, and in the southwest of Gansu Province, all of which have an average elevation of 3000–6000 m. Tibetan pigs are strongly adapted to the natural environment of the plateaus, including tolerance to cold and hypoxia [19]. As a result of economic development, the breeding mode has been transformed from free-grazing to large-scale feed-lots. Despite the large amounts of data on *Cryptosporidium* spp. in pigs worldwide, there have been very few studies on the occurrence and distribution of *Cryptosporidium* spp. in pigs in China. At present, there is no information on the prevalence of *Cryptosporidium* spp. in Tibetan pigs in Tibet, China. Therefore, the aim of this study was to examine the prevalence, identity, and molecular characteristics of *Cryptosporidium* spp. in three different pig breeds in Tibet and Henan, China, and to estimate their zoonotic potential.

## Methods

### Sample collection

**Fig. 1:**
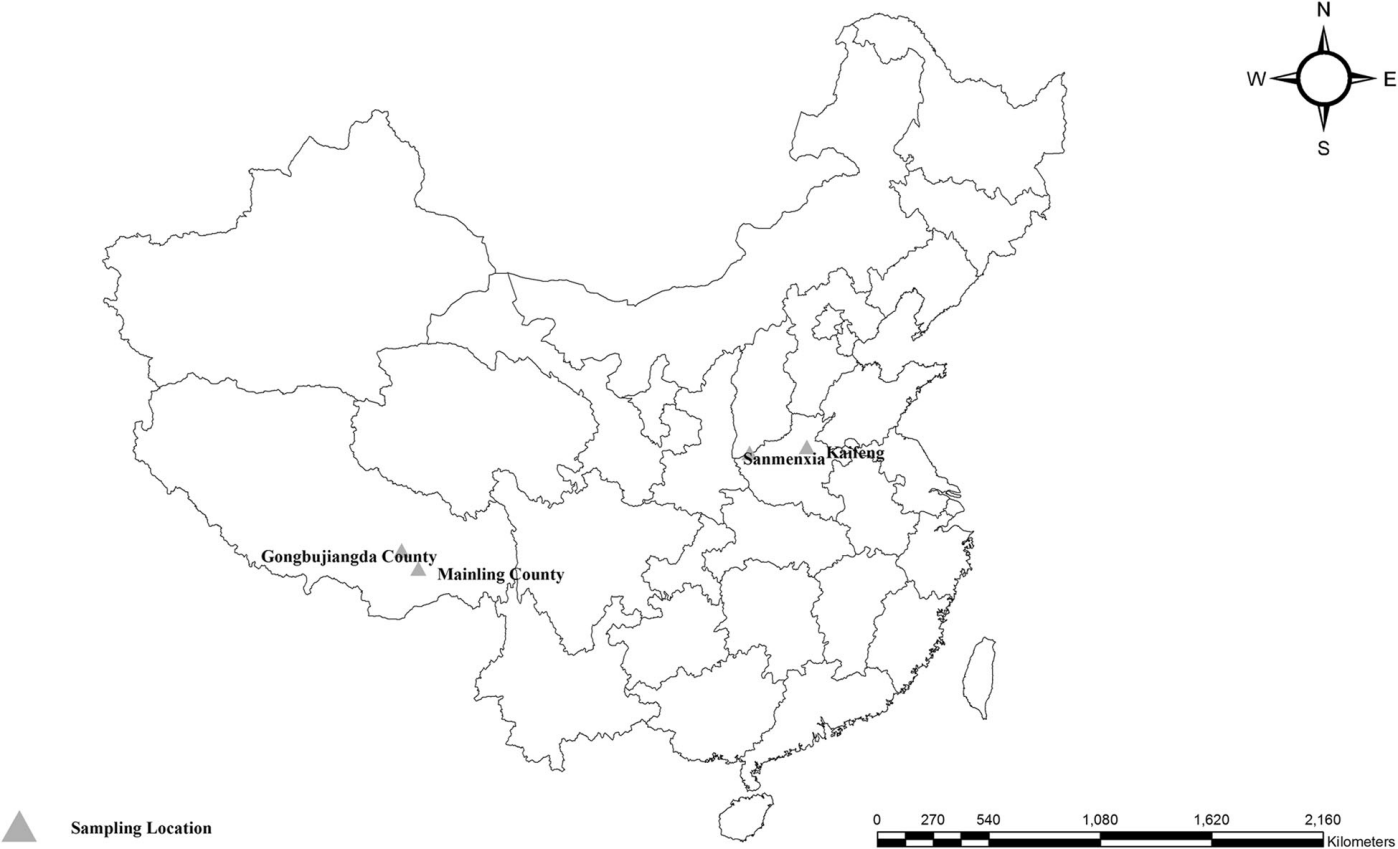
Map of sampling locations in China. Tibet is relatively high elevation, while Henan is located on the plains

From March to June 2016, a total of 1089 fresh faecal samples, each of ~ 20 g, were collected from four intensive farms in four areas of China, and each farm was visited only once (Fig. 1; Table 1). The samples were numbered and the sample details were recorded, including sampling time, geographic information and growth stage. During specimen collection, only the inner portion of each faecal sample was collected to ensure no environmental contamination. Each fresh faecal sample was separately collected into sterile gloves before adding 2.5% potassium dichromate and then placed into containers filled with ice packs and immediately transported to the laboratory. Upon arrival, each specimen was first treated by Sheather’s sugar flotation technique, and then examined by microscopy to detect *Cryptosporidium* oocysts at a bright-field microscope with 100 × and 400 × magnification. All fecal specimens were stored at 4 °C prior to DNA extraction.

**Table 1:** PCR-based results showed the infection rates and distribution of *Cryptosporidium* species in pigs of different collection sites, breeds and age groups

Factors		No. positive/sample size	Prevalence (%) (95% CI)	*Cryptosporidium* species (no.)
Collection site	Gongbujiangda County, Tibet	0/180	0	/
	Mainling County, Tibet	3/434	0.69 (0–1.47)	*C. scrofarum* (3)
	Sanmenxia, Henan	1/246	0.41 (0–1.21)	*C. suis* (1)
	Kaifeng, Henan	19/229	8.30 (4.70–11.90)	*C. suis* (11), *C. scrofarum* (8)
Breed	Tibetan pig	3/614	0.49 (0–1.04)	*C. scrofarum* (3)
	Yunan Black pig	1/246	0.41 (0–1.21)	*C. suis* (1)
	Landrace pig	19/229	8.30 (4.70–11.90)	*C. suis* (11), *C. scrofarum* (8)
Age group	< 1 month	1/467	0.21 (0–0.63)	*C. suis* (1)
	1–2 months	21/482	4.36 (2.53–6.19)	*C. suis* (11), *C. scrofarum* (10)
	> 2 months	1/140	0.71 (0–2.13)	*C. scrofarum* (1)
Total		23/1089	2.11 (1.26–2.97)	*C. suis* (12), *C. scrofarum* (11)

### DNA extraction and nested polymerase chain reaction amplification

Prior to DNA extraction, fecal samples were washed with distilled water to remove the potassium dichromate. Subsequently, genomic DNA was extracted from approximately 200 mg of semi-purified product using an E.Z.N.A.R Stool DNA Kit (Omega Bio-Tek Inc., Norcross, GA, USA) as per the manufacturer’s instructions. The extracted DNA was stored in 200-μl volume of Solution Buffer (supplied with the kit) at − 20 °C until use.

*Cryptosporidium* species were identified by nested PCR amplification and sequencing of an approximately 840 bp fragment of the small subunit rRNA (SSU rRNA) gene, as decribed previously [20]. A PCR product of approximately 1325 bp was first amplified with primers F1: 5*′*-TTCTAGAGCTAATACATGCG-3*′* and R1: 5*′*- CCCATTTCCTTCGAAACAGGA -3*′*. The amplification was performed in 25 μl volume with 1 μl of each DNA sample in 2.5 μl 10 × PCR buffer, 2.5 μl deoxynucleotide triphosphates (2 mM each), 1.5 μl MgSO4 (25 mM), 0.5 μl each primer (25 μM), 16 μl double distilled water, and 0.5 μl KOD-Plus amplification enzyme (1 units/μl) (ToYoBo Co., Ltd., Osaka, Japan). The first PCR reaction consisted of an initial heating at 94 °C for 5 min, and 35 cycles of 94 °C for 45 s, 55 °C for 45 s, and 72 °C for 1 min followed by a final extension at 72 °C for 10 min. A secondary PCR product of about 840 bp was then amplified with primers F2: 5*′*-GGAAGGGTTGTATTTATTAGATAAAG-3*′* and R2: 5*′*-AAGGAGTAAGGAACAACCTCCA-3*′*. The PCR and cycling conditions were identical to the primary PCR. The secondary PCR products were visualized by staining with Golden View following 1% agarose gel electrophoresis.

### Sequence analysis

All of the secondary amplification products were sequenced in both directions on an ABI PRISM 3730 XL DNA Analyzer using a BigDye Terminator v3.1 Cycle Sequencing Kit (Applied Biosystems, Foster City, CA, USA). To ensure sequence accuracy, two-directional sequencing was used. To identify *Cryptosporidium* species, the resulting sequences were subjected to BLAST analysis against sequences in the GenBank database (http://blast.ncbi.nlm.nih.gov), and aligned using Clustal X 2.1 software (http://www.clustal.org/).

### Statistical analysis

The *Cryptosporidium* infection rates were evaluated using Regression Analysis in SPSS 22.0 software for Windows with 95% confidence intervals (CI), and probability leval (*P*) of < 0.05 were considered as statistically significant.

## Results

### Occurrence of *Cryptosporidium*

Firstly, only five positive samples (5/1089) were detected by microscopy, and then 23 positive samples (23/1089) indentified by PCR (Table 1). The total infection rate of *Cryptosporidium* spp. in 1089 fecal samples was 2.11% (95% CI 1.26–2.97). The overall *Cryptosporidium* infection rate of pigs in Tibet was 0.49% (3/614, 95% CI 0–1.04). The prevalence in Mainling County was 0.69% (3/434, 95% CI 0–1.47), while no *Cryptosporidium*-positive sample was found in Gongbujiangda County. The total infection rate of *Cryptosporidium* in Henan was 4.21% (20/475, 95% CI 2.40–6.02), and Kaifeng had a higher rate 8.30% (19/229, 95% CI 4.70–11.90) than Sanmenxia 0.41% (1/246, 95% CI 0–1.21) (*p* < 0.01). Among the three different pig breeds, the infection rates of *Cryptosporidium* in local pig breeds including Tibetan pigs and Yunan Black pigs were 0.49% (3/614, 95% CI 0–1.04) and 0.41% (1/246, 95% CI 0–1.21), both lower than 8.30% (19/229, 95% CI 4.70–11.90) in imported Landrace pigs (*p* < 0.01) (Table 1).

The results showed that the prevalence of *Cryptosporidium* in weaned piglets (1–2 months) was 4.36% (21/482, 95% CI 2.53–6.19), which was significantly higher than the rates observed in pre-weaned piglets (< 1 month; 0.21%, 1/467, 95% CI 0–0.63) (*p* < 0.01) and finished pigs (> 2 months; 0.71%, 1/140, 95% CI 0–2.13) (*p* < 0.05) (Table 1). Among the 1–2 months old pigs infected with *Cryptosporidium*, there were three Tibetan pigs, one Yunan Black pig and 17 Landrace pigs. Interestingly, there was only one Landrace pig infected with *Cryptosporidium* in each of the other two ages.

### Species distribution

Sequence analysis of the SSU rRNA gene fragment revealed the presence of two *Cryptosporidium* species: *C. suis* (*n* = 12) and *C. scrofarum* (*n* = 11), and also showed 100% nucleotide identity to a pig-derived sequence (GenBank accession number: GU254174) and pig-derived sequences (GenBank accession numbers: KU668895 and KP704557) respectively. All sequence data has been deposited in the GenBank database under accession numbers MH174659, MH174663, MH178034, and MH178036.

In Henan, two *Cryptosporidium* species were indentified, including 12 *C. suis*-positive samples and 8 *C. scrofarum*-positive samples, while only 3 *C. scrofarum*-positive samples were found in Tibet. There were many *Cryptosporidium* -positive samples in imported Landrace pigs, including 11 *C. suis*-positive samples and 8 *C. scrofarum*-positive samples, but fewer *Cryptosporidium*-positive samples in local pig breeds, including 3 *C. scrofarum*-positive samples in Tibetan pigs and one *C. suis*-positive sample in Yunan Black pigs. In addition, *C. suis* was only indentified in pigs aged < 1 month old (*n* = 1) and 1–2 months old (*n* = 11), and *C. scrofarum* was only indentified in pigs aged 1–2 months old (*n* = 10) and > 2 months old (n = 1).

## Discussion

Among the 23 *Cryptosporidium*-positive samples identified by PCR, only 5 were detected by microscopy, which may be caused by the low concentration of *Cryptosporidium* oocyst in fecal specimens. In this study, the *Cryptosporidium* prevalence in Henan was 4.21%, which is lower than that in many reported areas of China, such as Guangdong (8.33%, 6/72), Zhejiang (14.52%, 18/124), Yunnan (23.00%, 46/200), Shanghai (34.44%, 800/2323), Heilongjiang (55.75%, 63/113), and other regions, but only higher than Shaanxi (3.29%, 44/1337) (Table 2) [21–27]. Surprisingly, the infection rate of *Cryptosporidium* in Tibet is lower than in any reported areas (Table 2). The present study also indicated that the *Cryptosporidium* infection rates in both local pig breeds were lower than that in imported pig breed. The observed *Cryptosporidium* prevalence rates of local pig breeds, including 0.49% in Tibetan pigs and 0.41% in Yunan Black pigs, were also apparently lower than that reported in some foreign pig breeds in North America (17.85–55.74%), Europe (5.36–74.67%), and Asia (59.68–71.43%), and in wild boar in the Czech Republic (16.68%) and Spain (16.75%) (Table 3) [6, 28–39]. Many factors, including management system, specimen size and diagnostic technique, may be also responsible for the differences in the prevalence of *Cryptosporidium* among different pig breeds and different geographic areas.

**Table 2:** Distribution of *Cryptosporidium* species/genotypes amongst pigs in China

Collection site	No. positive/sample size	Prevalence (%) by microscopically or others (95% CI)	No. positive/sample size	Prevalence (%) by PCR (95% CI)	*Cryptosporidium* species (no.)	Reference
Zhejiang			18/124	14.52 (8.23–20.80)	*C. scrofarum* (18)	Zou et al. (2017) [21]
Guangdong			6/72	8.33 (1.79–14.87)	*C. scrofarum* (6)	Zou et al. (2017) [21]
Yunnan			46/200	23.00 (17.12–28.88)	*C. scrofarum* (46)	Zou et al. (2017) [21]
Shaanxi			44/1337	3.29 (2.33–4.25)	*C. suis* (42), *C. scrofarum* (2)	Lin et al. (2015) [22]
Heilongjiang	9/568	1.58 (0.55–2.61)	63/113	55.75 (46.45–65.05)	*C. suis* (22), *C. scrofarum* (23) *C. suis* + *C. scrofarum* (18)	Zhang et al. (2013) [23]
Shanghai and Zhejiang, Shaoxing			79/208	37.98 (31.33–44.63)	*C. suis* (14), *C. scrofarum* (65)	Yin et al. (2013) [24]
Zhejiang, Shaoxing			6/24	25.00 (6.32–43.68)	*C. scrofarum* (6)	Yin et al. (2011) [25]
Shanghai			10/70	14.29 (5.88–22.69)	*C. scrofarum* (10)	Yin et al. (2011) [25]
Shanghai	800/2323	34.44 (32.50–36.37)			*C. suis* (57), *C. scrofarum* (6) *C. suis* + *C. scrofarum* (6)	Chen et al. (2011) [26]
Henan	111/1350	8.22 (6.76–9.69)	108/1350	8.00 (6.55–9.45)	*C. suis* (94), *C. scrofarum* (14)	Wang et al. (2010) [27]
Total	920/4241	21.69 (20.45–22.93)	380/3498	10.86 (9.83–11.90)	*C. suis* (229), *C. scrofarum* (196) *C. suis* + *C. scrofarum* (24)

**Table 3:** Distribution of *Cryptosporidium* species/genotypes amongst pigs worldwide

Collection site	No. positive/sample size	Prevalence (%) by microscopically or others (95% CI)	No. positive/sample size	Prevalence (%) by PCR (95% CI)	*Cryptosporidium* species (no.)	Reference
Japan	112/344	32.56 (27.58–37.53)	37/62	59.68 (47.12–72.24)	*C. suis* (13), *C. scrofarum* (16), *C. suis* + *C. scrofarum* (8)	Yui et al. (2014) [6]
central Vietnam	28/193	14.51 (9.49–19.52)	10/14	71.43 (44.36–98.50)	*C. suis* (8), *C. scrofarum* (2)	Nguyen et al. (2013) [28]
Danish	350/856	40.89 (37.59–44.19)	56/75	74.67 (64.59–84.74)	*C. suis* (18), *C. scrofarum* (38)	Petersen et al. (2015) [29]
Austria	2/44	4.55 (0–10.95)	8/44	18.18 (6.32–30.04)	*C. suis* (2), *C. scrofarum* (3), *C. suis* + *C. scrofarum* (3)	Němejc et al. (2013) [30]
Australia			45/289	15.57 (11.37–19.78)	*C. suis* (13), *C. scrofarum* (32)	Johnson et al. (2008) [31]
The Czech Republic	6/231	2.60 (0.53–4.66)	39/231	16.88 (12.02–21.75)	*C. suis* (13), *C. scrofarum* (14), *C. suis* + *C. scrofarum* (12)	Němejc et al. (2013) [30]
The Czech Republic	194/1620	11.98 (10.39–13.56)	353/1620	21.79 (19.78–23.80)	*C. suis* (142), *C. scrofarum* (126), *C. suis* + *C. scrofarum* (82), *C. parvum* (1), *C. muris* (3)	Němejc et al. (2013) [32]
The Czech Republic^a^	0/193	0	32/193	16.58 (11.29–21.87)	*C. suis* (13), *C. scrofarum* (7), *C. suis* + *C. scrofarum* (12)	Němejc et al. (2012) [33]
The Czech Republic	87/413	21.07 (17.12–25.01)	69/413	16.71 (13.09–20.32)	*C. suis* (45), *C. scrofarum* (22), *C. muris* (2)	Kváč et al. (2009) [34]
The Czech Republic, South Bohemia	38/144	26.39 (19.10–33.67)	38/144	26.39 (19.10–33.67)	*C. suis* (2), *C. scrofarum* (21), *C. suis* + *C. scrofarum* (15)	Kváč et al. (2009) [35]
Poland	3/129	2.33 (0–4.96)	11/129	8.53 (3.64–13.41)	*C. suis* (1), *C. scrofarum* (8), *C. suis* + *C. scrofarum* (2)	Němejc et al. (2013) [30]
The Slovak Republic	0/56	0	3/56	5.36 (0–11.44)	*C. suis* (2), *C. scrofarum* (1)	Němejc et al. (2013) [30]
Canada, Prince Edward Island	163/633	25.75 (22.33–29.17)	113/633	17.85 (14.86–20.84)	*C. suis* (41), *C. scrofarum* (69), *C. parvum* (2), Mouse genotype (1)	Buduamoako et al. (2012) [36]
Canada, Ontario	54/122	44.26 (35.32–53.20)	68/122	55.74 (46.80–64.68)	*C. scrofarum* (26), *C. parvum* (38)	Farzan et al. (2011) [37]
Spain, Zaragoza	32/142	22.54 (15.58–29.49)	26/142	18.31 (11.87–24.75)	*C. suis* (10), *C. scrofarum* (16)	Suárezluengas et al. (2007) [38]
Spain, Galicia ^a^			35/209	16.75 (11.64–21.85)	*C. suis* (5), *C. scrofarum* (19), *C. parvum* (3)	Garcíapresedo et al. (2013) [39]
Total	1069/5120	20.88 (19.77–21.99)	943/4376	21.55 (20.33–22.77)	*C. suis* (328), *C. scrofarum* (420), *C. suis* + *C. scrofarum* (134), *C. parvum* (44), *C.muris* (5), Mouse genotype (1)	

^a^The samples from these two studies came from wild boars

*Cryptosporidium* infections are common in pigs and have been found in all age groups worldwide. However, many studies have shown that the highest rates of *Cryptosporidium* infection occur in weaned piglets (1–2 months), with the infection rates ranging from 20.63 to 83.72% indentified by PCR [6, 23, 26, 27]. In this study, the prevalence of *Cryptosporidium* in weaned piglets (1–2 months) was at a lower level, but still consistent with the studies of others that weaned piglets (1–2 months) was higher than that in other two age groups. Therefore, although the age distribution of *Cryptosporidium* infection rates in pigs has not been clearly concluded up to now, we can generally assume that the rate of *Cryptosporidium* infection in pigs aged 1–2 months is greater than that of other age groups. It is difficult to explain why previous studies have found that conventionally-reared piglets < 1 month of age are reliably infected under the conditions examined. The age distribution of *Cryptosporidium* infection may also be affected by a number of other factors, including changes in the intestinal environment of the animals caused by diet or age.

Many studies have shown that *C. suis* and *C. scrofarum* appear to be the main pig-adapted species, and some other *Cryptosporidium* species were also identified in pigs, sometimes with mixed infections. Oddly, there was no mixed infection in our study. In this study, *C. suis* and *C. scrofarum* were both found in Henan, which was the same as that in Shaanxi and other regions that have been reported [22–24, 26, 27] (Table 2), while *C. scrofarum* was only identified in Tibet, which was the same as that in Guangdong and other regions that have been reported [21, 25] (Table 2). So far, mixed infections of *C. suis* and *C. scrofarum* were only reported in Heilongjiang and Shanghai, China [23, 26] (Table 2). *C. suis* and *C. scrofarum* were detected in local Yunan Black pigs and Tibetan pigs in this study, respectively. However, *C. suis* and *C. scrofarum* were fairly common, and mixed infections of *C. suis* and *C. scrofarum* were reported in some foreign breeds in Japan, Austria, The Czech Republic, and Poland [6, 30, 32, 33, 35] (Tables 3). Interestingly, *C. parvum*, *C. muris*, and mouse genotype strains have also been reported in pigs in several countries [32, 34, 36, 37, 39] (Tables 3). In addition, our results also showed that 12 samples from one pre-weaned piglet (< 1 month) and 11 weaned piglets (1–2 months) contained *C. suis*, while 11 samples from 10 weaned piglets (1–2 months) and one finished pig (> 2 months) contained *C. scrofarum*. These findings are consistent with a previous report showing that the different *Cryptosporidium* species are age-specific in pigs, and piglets are more susceptible to *C. suis* infection while older pigs are more susceptible to *C. scrofarum* [24]. There are many factors leading to differences in the identification of single or mixed infection, with insufficient testing accuracy being a major cause. A comparative study found that one *Cryptosporidium*-positive sample was typed as *C. muris* on the basis of Sanger sequencing but was identified as a *C. muris* and *C. tyzzer* mixed infection by high-throughput sequencing, which has a superior depth of coverage, especially for mixed infections in clinical samples [40]. Another study found that *C. suis* and *C. scrofarum* in mixed infections were successfully identified using Illumina sequencing technology [41]. However, to improve identification rates of *Cryptosporidium* species or genotypes in mixed-infection samples, more sensitive tools with greater resolution are required.

## Conclusions

In conclusion, although the levels of *Cryptosporidium* infection were lower in three different pig breeds in Tibet and Henan, especially in Tibetan pigs and Yunan Black pigs, the two indentified *Cryptosporidium* species, including *C. suis* and *C. scrofarum* are both zoonotic. Importantly, this is the first epidemiological investigation of the prevalence and risk factors of *Cryptosporidium* in Tibetan pigs from Tibet. At present, there is no effective drug treatment or vaccine for porcine cryptosporidiose. Therefore, measures such as strengthening the breeding management of pigs and improving the sanitary and safe disposal of pig feces are needed to avoid the spread of pathogens. In addition, further molecular epidemiological surveys of *Cryptosporidium* in pigs, humans, and other animals are also needed to better elucidate the mode and risk of transmission.

## Abbreviations

PCR: Polymerase chain reaction
SSU rRNA: Small subunit rRNA
